# Exploring the organisational structure of networks for exercise oncology provision: a social network analysis of OnkoAktiv

**DOI:** 10.1186/s12913-023-09572-8

**Published:** 2023-05-27

**Authors:** Annelie Voland, Maximilian Köppel, Stefan Peters, Joachim Wiskemann, Hagen Wäsche

**Affiliations:** 1 National Center for Tumor Diseases (NCT), Im Neuenheimer Feld 460, 69120 Heidelberg, Germany; 2 Institute of Sports and Sport Science, Heidelberg University, Heidelberg, Germany; 3 Deutscher Verband Für Gesundheitssport Und Sporttherapie E.V. (DVGS), Vogelsanger Weg 48, 50354 Hürth-Efferen, Germany; 4 Institute of Sports and Sports Science (IfSS), Karlsruhe Institute of Technology (KIT), Engler-Bunte-Ring 15, 76131 Karlsruhe, Germany

**Keywords:** Social network analysis, Exercise oncology, Cancer, Inter-organisational collaboration, Network governance, Network forms, Integrated care

## Abstract

**Background:**

Structured exercise programs provide considerable health benefits for cancer patients. Therefore, various OnkoAktiv (OA) networks were established in Germany with the aim to connect cancer patients with certified exercise programs. However, knowledge about the integration of exercise networks into cancer care systems and conditions of interorganisational collaboration is lacking. The aim of this work was to analyse the OA networks to guide further network development and implementation work.

**Methods:**

We used methods of social network analysis within a cross-sectional study design. Network characteristics were analysed such as node and tie attributes, cohesion and centrality. We classified all networks into their level of organisational form in integrated care.

**Results:**

We analysed 11 OA networks with 26 actors and 216 ties on average. The smallest network counted 12 actors/56 ties, the largest 52/530. 76% of all actors operated within the medical/exercise sector, serving 19 different medical professions. In smaller “linkage” networks, several individual professionals were linked “from service to service”, whereas the more integrated networks revealed a core-periphery-structure.

**Discussion:**

Collaborative networks enable the involvement of professional actors from different operational fields. This study provides an in-depth understanding of underlying organisational structures that provides information for further development of exercise oncology provision.

**Trial registration:**

Not applicable, as no health care intervention was performed.

**Supplementary Information:**

The online version contains supplementary material available at 10.1186/s12913-023-09572-8.

## Background

A large body of evidence supports the significant positive effects of physical activity and exercise in cancer patients and survivors [1]. Several cancer- and cancer-treatment related side effects such as cancer related fatigue, anxiety, secondary lymphedema or functional disabilities can be prevented or diminished by exercise therapy [2]. Moreover, long-term observational studies have shown a 40–50% decrease in cancer-related mortality for breast-, colon- and prostate cancer in physically active patients [3, 4]. However, the current situation of exercise oncology provision in Germany is fragmentary and heterogenous. Most exercise programs and networks are tailored to the needs of the individual region or institution. They differ from one region to another in many aspects. Further, there is not much knowledge about the integration of exercise provision into cancer care systems and collaborations between care services [5]. Especially the development of exercise-oncology clinical pathways [6], based on collaborations across different care sectors remain an important aspect for exercise care integration [7]. Specific knowledge about methods and models on pooled administration, funding and service delivery would be essential for comprehensive exercise implementation, as recommended by the American College of Sports Medicine (ACSM). The ACSM engages health care professionals (HCPs) to screen, advice and refer cancer patients into different types of exercise programs according to their needs [6]. To serve the demand of an exercise care in Germany, the nation-wide network OnkoAktiv was established. It aims to connect clinical structures (including their stakeholders) with community-based exercise programs (e.g. gyms, fitness centers) to enable patient referral into cancer-specialized exercise programs [8].

To date, 15 regional OnkoAktiv networks, coordinated through regional OnkoAktiv centers, have been established as “sub-networks” of OnkoAktiv. Each regional OnkoAktiv network works independently under the “OnkoAktiv umbrella” and integrates the OnkoAktiv instruments and processes (e.g. screening, exercise consultation, referral processes) into their local clinical context. Regional OnkoAktiv networks are managed by OnkoAktiv coordinators.

The collective OnkoAktiv network has been growing fast over the years, although a structural and research-based network evaluation has not been applied yet. There is currently no systematic approach to either record existing inter-organisational collaboration within physical activity networks in oncological settings nor to measure exercise care integration into cancer care systems [9]. Accordingly, the World Health Organisation (WHO) states within the global action plan on physical activity, that collaboration across and between all stakeholder at all levels is needed to realize the multiplicative benefits of a more physically active world [10]. Further, the emergence of clinical pathway of exercise care show promising results in regard to exercise integration, however, current findings reveal major challenges with HCP collaborations and referral processes from clinical to exercise settings [5]. In summary, the development of collaborative networks in exercise oncology can be rated as highly important for the integration of exercise into cancer care. Therefore, this work aimed better understand the collaborative structures of the OnkoAkiv networks to enhance further network development. The analysis followed two main goals:

- 1. To describe each OnkoAktiv network in regard to their major network characteristics and classify each network into their developmental stage of organizational forms.
- 2. To define implications and tasks for demand-oriented network implementation and further development in exercise oncology.

### Theoretical framework for the analysis of the OnkoAktiv networks

Social network analysis (SNA) allows to describe, explore and understand social systems [11–13]. For this reason we used SNA in our study on OnkoAktiv networks. There are various methodological concepts in SNA such as the analysis of node and tie attributes, cohesion and centrality (see Wäsche et al. [13] for further explanations). For the purpose of this study, we used the following concepts [14, 15].

First, we analysed all OnkoAktiv networks as ego-networks, where the individual network, from an ego´s view, is in the focus of analysis. To explore the characteristics of the OnkoAktiv networks, node and tie attributes were identified. This involves the task and profession of network actors (node attributes) as well as their types of relations (tie attributes). With regard to node attributes, the actor’s task and profession were collected, which reveal information about the network actors. This implies, for example, the profession “doctor”. A doctor is associated with a set of tasks and attitudes that doctors “should do” (25). Tie attributes represent actions and relations between actors such as collaborations in regard to patient care or financing services. They contribute to a better understanding of interaction pattern and allow to characterize the set-up and interaction patterns of OnkoAktiv networks.

To shed light on network cohesion, we used the parameter average degree. It reports the mean number of ties of each actor, indicates the overall cohesiveness of OnkoAktiv networks and enables a structural comparison.

Centrality defines the position of an actor within a network. We utilized two centrality measurements. Degree centrality identifies central, well connected and important actors in OnkoAktiv networks besides ego. Betweenness centrality describes the extent to which an actor bridges two parts of a network. Actors with high betweenness, also called brokers, have high control over the flow of information and resources in the network [14, 16–20]. According to current research [21], centrality can be a valid measure in ego-centric networks. Therein, centrality reflects an alter´s level of embeddedness in ego´s network and this in turn builds for example trust and willingness to engage with this actor (even though it is a very subjective point of view). Therefore, how the OA coordinator perceives a structural position of an actor is a key factor to exchange resources or information.

To analyse the macro-structure, network core and periphery can be considered [13, 22]. The nodes (actors) of a network are partitioned in two groups: the well-connected core and the nodes in the periphery of a network. The continuous model defines, in which each node is assigned a measure of coreness that presents the position of a node in relation to the estimated network center [22].

To classify networks in health and social care, different models of organisational forms have been identified within the literature [23–25]. Such models position social networks along a continuum of organisational forms and support the classification of OnkoAktiv networks into their level of network maturity. For our analysis, we applied the continuum by Leutz [25], in which three levels “linkage”, “co-ordination” and “full integration” have been utilized. In theory, the form “linkage” is associated with a number of stakeholders (e.g. HCPs) that are losely connected but understand on both sides, who needs to take care of what service, how costs are spread and who receives the benefits. The network structure is informal and flat. In co-ordinated networks, defined networks structures and network managers are installed to coordinate care services across the care system. However, co-ordinated networks are operating mostly on existing and separate structures, also financial and clinical responsibilities remain separate (e.g. individual health care coverage for each service). Such networks provide cross-institutional collaboration but without any bounding contract. The highest level “full integration” builds a new care service in which resources such as finances, staff or expertise are pooled. Multidisciplinary teams define common benefits and they control the new program as the “whole”.

**Fig. 1:**
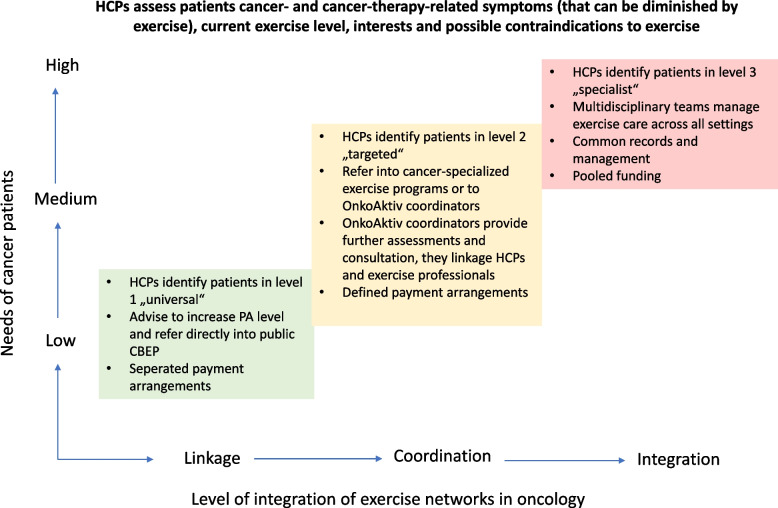
Tasks for HCPs in relation to the level of patients’ needs and the organisational level of network integration adapted by Leutz [25]

According to Leutz [25], the demand of care integration into the system is based on patients’ needs such as complexity of disease, level of impairments or cancer- and cancer-therapy-related symptoms. For the categorisation of patients, the “prehab-/rehab-triangle” by the Macmillan Cancer Support in the United Kingdom can be applied. The triangle distinguishes patients between universal, targeted and specialist in which patients can move up or down if, their disease diminish or progresses. Universal exercise programs are applicable for anyone with cancer, targeted programs are designed for people with cancer with acute chronic symptoms of their disease and/or long-term conditions. Specialised programs are applicable to patients with cancer who have complex needs, severe physical impairments or disabilities, unstable conditions or low physical activity levels [26]. The dimensions of patients’ needs (mild, moderate, severe) have been also described by Leutz [25]. Figure 1 illustrates the level of patients’ needs in relation to the organisational level of integration by Leutz [25] and the defined tasks for HCPs according to the ACSM recommendations [6].

## Methods

### Sampling, data collection and study instruments

We applied an ego-centric network analysis within a cross-sectional study design which elaborates the existence of collaborations from the OA coordinator’s view. The OA coordinator was the leading professionals of each regional OnkoAktiv network and the interview participant in this study. After agreement of the consent of research, the interviews were held online via the video conference platform Zoom, based on a standardized questionnaire. Our questionnaire followed the methodological process of egocentric SNA by Borgatti [14] and Perry [21]. First, we asked OnkoAktiv coordinators as our “ego” to list their most relevant contact persons including their job position and the organisation or unit they represent via name generators. For the application of name generators, we pre-defined deductive categories (medicine/exercise science, charitable foundations, associations in the fields of physical activity/exercise, cancer associations, university, health care insurances, local organisations). We then applied name and relations interpreters including information about type (patient-related, influential, financial, public communication-related) and importance of relations, duration of collaborations and single important positions of individuals. Finally, we asked ego about all alter-alter-connections to construct a full network matrix. The study protocol has been approved by the ethics committee of the medical faculty at the University Heidelberg (S-942/2021 and S-915/2019).

### Network measurements

Based on the theoretical framework in the introduction about SNA measurements and concepts, we applied the following structural parameters [13]:

• Node and tie attributes: task and profession of actors, tie distribution
• Cohesion: number of nodes and ties, average degree
• Centrality: degree and betweenness centrality
• Macrostructure: core-periphery-structures

Further, we classified all networks into the continuum of organizational forms by Leutz [25].

Those network measurements helped to describe each OnkoAktiv network in regard to their major network characteristics. Further, the visualisation of specific network measurements through network graphs enabled the classification of each network into their developmental stage of organizational forms.

Although we applied an ego-centric network analysis, full network measurements such as centraliy have been applied. Clearly, the most central actor in ego-centric networks is ego, however, degree centrality also discovers actors that are central within a given network besides ego. The same counts for betweenness centrality. We were interested in other influential actors next to ego, that have the power to control network flows such as information or resources.

### Data analysis

For data management, descriptive statistics, and computation of network measurements, we used the programs UCINET 6 for Windows – Version 6.730 and Microsoft Word Excel 2016. All networks were symmetrised and calculated as undirected, dichotomous networks. We defined the largest network A as our “benchmark” network to compare the OnkoAktiv networks to each other. The categorisation of organisational forms was based on the classification parameter in Table 1.

**Table 1:** Parameters for classification into organizational forms adapted from Leutz [25]

	**Linkage**	**Co-ordinated**	**Full integration**
Structural typology	Single actors within existing services are linked “service to service”	Single actors are coordinated by network managers (from within or outside the network)	A new program/service has been created with pooled benefits and recourse, sharing costs and defined tasks. Multidisciplinary teams control jointly all perspectives of the new service
Actor responsibilities	Screen, inform and refer patients to “other services” within the care system, responsibilities are separate	Managers share clinical information, manage transitions, coordinate benefits and the sequence of services (“care management”) Network actors/groups not bounded by any binding contract, responsibilities remain separate	Multi-disciplinary professional teams with joint clinical and contractual responsibilities Apply case management
Administrational body	None	Administration through elected network members	Managed through an individual, neutral administrative body
Patients’ needs	Universal	Targeted	Specialist

### Data visualisation

For data illustration, the visualization software Gephi (Version 0.9.2) was deployed. We applied the “Yifan Hu” algorithm for all networks. Depending on the interest of visualization, we changed node size and color for better visualization (see captions of graphs).

## Results

### Descriptive statistics

We held 11 interviews with OnkoAktiv coordinators. Four networks (A, C, G, H) had been an OnkoAktiv member for more than 3 years, the other seven networks had been network members for less than 3 years at the time of data collection. A full overview of network can be seen in Table 2.

**Table 2:** Overview on OnkoAktiv network measurements ordered by network size

Networks	G	I	L	M	D	H	J	E	B	C	A
# of nodes	12	16	17	19	19	23	26	32	33	39	52
# of ties	56	92	68	148	166	216	190	224	202	478	530
Average Degree	4.7	5.75	4.0	7.8	8.7	9.4	7.3	7.0	6.1	12.3	10.2
**Tie attributes**											
Patient-related [%]	50.0	56.3	64.7	84.2	63.1	69.6	65.4	65.6	72.7	38.5	52.9
Influence [%]	58.3	68.8	58.8	36.8	52.6	47.8	69.2	75.0	45.5	82.1	82.4
Finances [%]	16.7	25.0	35.3	21.1	26.3	39.1	34.6	25.0	9.1	20.5	33.3
Public communication [%]	50.0	81.3	70.6	57.9	36.8	69.6	23.9	56.3	75.8	25.6	35.3
Years of collaboration [Avg]	6.8	4.0	4.9	3.9	1.0	6.3	4.8	1.8	2.1	5.1	7.0
Importance; 1–10 [Avg]	7.0	5.9	7.2	6.3	7.3	7.0	4.8	8.4	6.7	6.5	5.7
**Core nodes**											
# of core nodes [n]	7	2	3	11	13	8	7	5	3	15	6
[%] of total nodes	58	13	18	58	68	35	27	16	9	39	12

### Node and tie attributes: what are the tasks and professions of the network actors?

The categorization of actors into pre-defined sectors showed that 76% of all actors held a professional position within the medical/exercise science sector. However, smaller networks indicated higher numbers (up to 100%) of medical professions compared to larger networks. Subsequently, larger networks showed more diversity in their professional distribution.

**Fig. 2:**
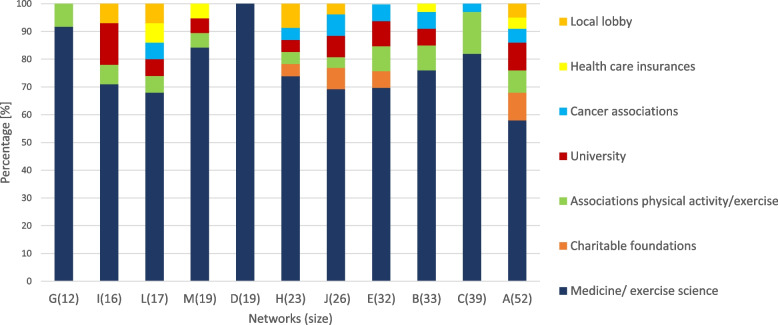
Distribution of actor-categories across networks in percentage; networks are sorted according to network size from left (G = smallest network) to right (A = largest network)

As seen in Fig. 2, network A was the only network that involved all pre-defined categories of operational positions. A detailed analysis of the medical/exercise science sector showed 19 different professions with the highest number of actors in exercise science/ sports medicine (M = 8; min = 2; max = 18). Thereafter, most collaborations existed with oncologists, clinical directors, gynecologists, cancer rehabilitation and physiotherapists across all networks. The OnkoAktiv networks included 5–9 different medical health care professions regardless of their network size. A detailed analysis of medical professions in number of actors and percentages can be found in Supplement 1.

Each OnkoAktiv network had one to three subjectively perceived very important network actors, which all held medical-related professions. Most important actors in the three smallest networks (G, I, L) were clinical directors, leading clinical nurses and oncologists and leading exercise scientists. The professions of the most important actors in the three largest networks (A, C, B) were equal to the professions of the most important actors in the smaller networks. However, perceived leaders in larger networks came from different regions across Germany, whereas leaders in smaller networks worked in the same organisation as the interviewee.

Analysing the distribution of network ties, we found no consistent pattern across types of ties (patient-related, influence, finances and public-communication-related). However, financial ties showed the lowest incidence on average (see Table 2).

### Cohesion: number of nodes and ties, average degree

**Fig. 3:**
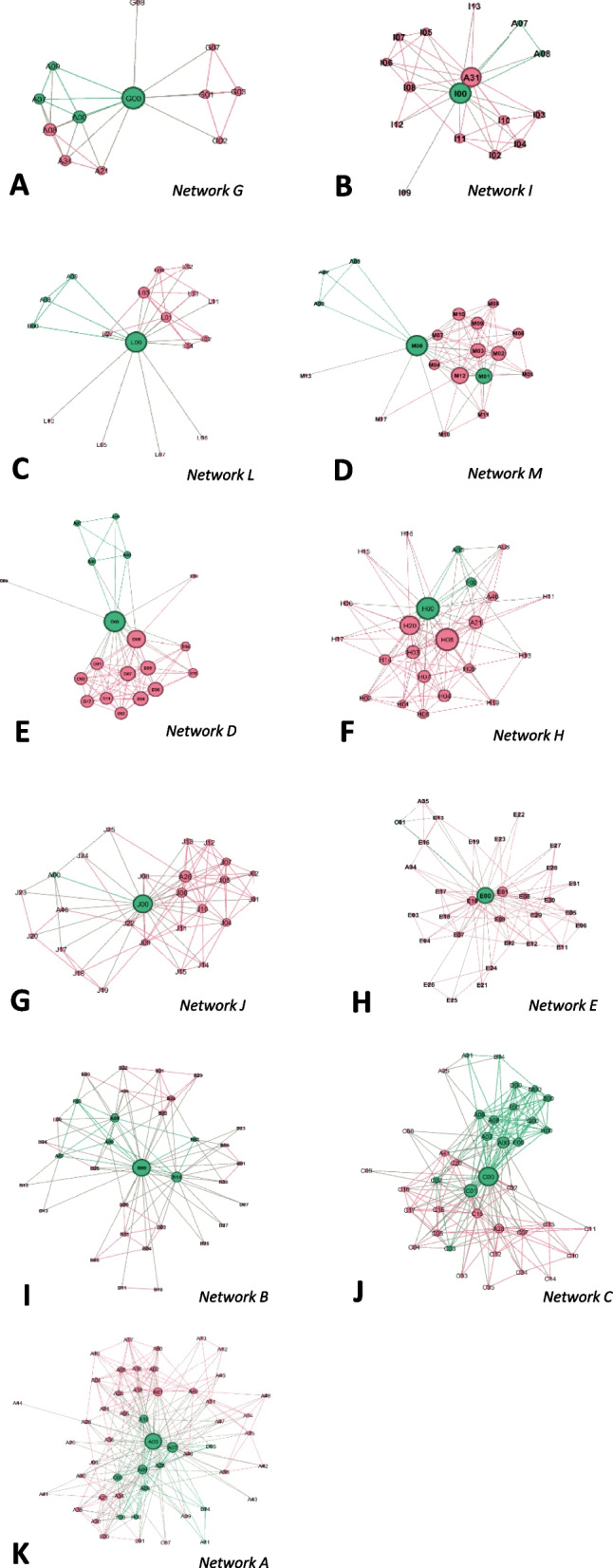
All OnkoAktiv networks sorted according to their network size (sub-figures **A**-**K**); nodes represent network actors; green color: member of OnkoAktiv; red color: other nodes; size of nodes indicates the degree of each actor; links represent a collaborative relationship

The network sizes (total number of actors) ranged from *n* = 12 (network G) to *n* = 52 (network A) with an average number of actors of *n* = 26.2 (SD = 11.3). The total number of indirect ties ranged from *n* = 56 (network G) to *n* = 530 (network A), with an average number of ties of *n* = 215.5 (SD = 147.2). The mean average degree of all actors in the networks was 7.6 (SD = 2.4), with the highest average degree of 12.3 (network C) and the lowest of 4.9 (network L). Figure 3 shows all OnkoAktiv networks, sorted according to their network size. The network visualizations revealed that smaller networks tend to connect different groups with high cohesion, whereas larger networks build tight connected core and loose peripheral structures.

### Centrality: who are actors in the center of the network?

For the comparison of OnkoAktiv networks, the largest network A (*n* = 52 nodes), network H with the median size (*n* = 23 nodes) and the smallest network G (*n* = 12 nodes) will be reported as exemplary networks. Within our benchmark network A, degree centrality ranged from 4 to 51, with five nodes (A00, A01, A07, A08, A10) having more than 20 ties. Next to Ego (A00), A07 (119.8) and A08 (73.7) showed the highest betweenness centrality. In network H, degree centrality ranged from 3 to 22 ties. The highest degree centrality was around 55% lower than in network A. Two nodes (H00, H08) held more than 20 ties each. Ego (H00) and H08 revealed the highest betweenness centrality (both 44.61). The analysis of network G displayed a range of degree centrality from 1 to 11. The highest degree was around 80% lower than in network A. Besides ego (G00), all nodes held 6 or less ties respectively, which is less than 50% of ties compared to network A and H. Ego showed the highest betweenness centrality (35.2), all other nodes revealed a betweenness of < 1.

### Macrostructure of OnkoAktiv networks

Based on the continuous core-periphery model [22] the number of core nodes in all OnkoAktiv networks ranged from 2 to 15 (M = 7.3; SD = 4.0). The number of core nodes did not increase with the total number of actors. Further, core nodes in smaller networks were either located only in ego´s organisation or the core split in two parts of different groups with ego as broker. In larger networks, the core operated on an inter-organisational network level, with actors from different regions and institutions in several important positions, like leading exercise scientists and clinical directors.

### Network classification: continuum of organizational forms

Based on the classification criteria described earlier (see Table 1), the analysis and visualization showed a systematic growth of structural maturity and level of exercise care integration. Although, OnkoAktiv networks presented some types of hybrid variations.

#### Linkage

**Fig. 4:**
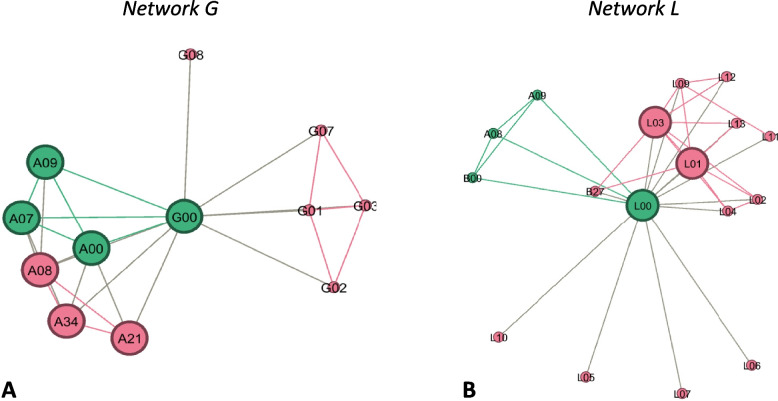
Example of “Linkage” (sub-figure **A**: network G; sub-figure **B**: network L); nodes represent network actors; green colour: member of OnkoAktiv; red colour: other nodes; large nodes indicate the network core; links represent a collaborative relationship

We categorized networks G, I, L, M and D as linkage [25]. See Fig. 4 for two examples in the category linkage. Within linked networks, most actors were connected to each other in an unorganized manner “from service to service”. The OnkoAktiv coordinator (ego) sit between two parts of the network and linked some single nodes from the periphery. The core was shifted to one side of the network with ego sitting on its edge. Overall number of nodes and ties was low compared to more integrated networks. Nevertheless, individual network components showed higher levels of intra-connectivity between actors but not any or just a few cross-sectional ties between separate components (see network G). Overall distribution of different professions was high, although most actors originated from regional institutions.

#### Co-ordinated networks

**Fig. 5:**
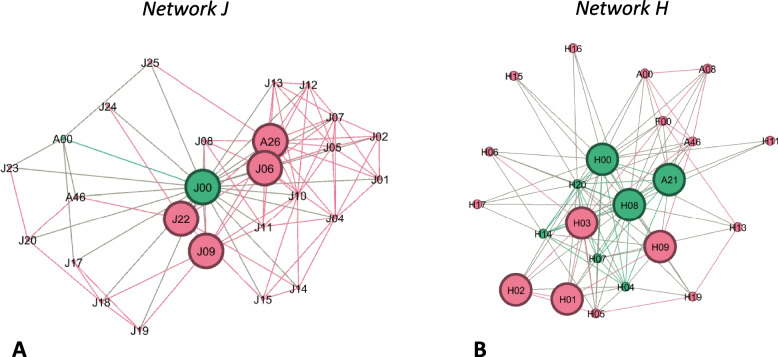
Examples of co-ordinated networks (sub-figure **A**: network J; sub-figure **B**: network H); nodes represent network actors; green colour: member of OnkoAktiv; red colour: other nodes; large nodes indicate the network core; links represent a collaborative relationship

As seen in Fig. 5, networks H and J could be classified as co-ordinated networks. Departments and network components were cross-linked (e.g. network J: A00-J25-A26) and multiple nodes were in similar structural positions, having many ties to the same actors (e.g. A26/J06/J10). Core nodes operated in different clinical professions and spread across institutions. Most actors were located in the medical sector (69–74%).

#### Full integration

**Fig. 6:**
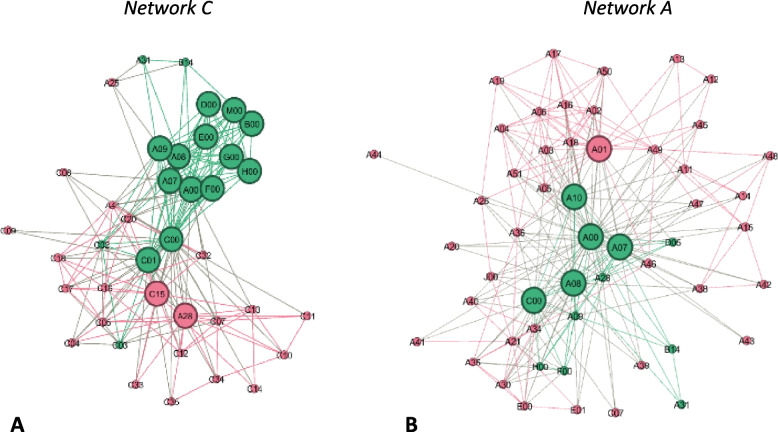
Example of more integrated networks as hybrid-version (sub-figure **A**: network C; sub-figure **B**: network A); nodes represent network actors; green colour: member of OnkoAktiv; red colour: other nodes; large nodes indicate the network core; links represent a collaborative relationship

We ranked networks E, B, C and A as full integration, although they also showed pattern of co-ordinated network structures as hybrid variations. Figure 6 illustrates two OnkoAktiv networks (C, A) as example for hybrid versions. The full integrated networks revealed an enlarged network size (*n* > 30 nodes; *n* > 200 ties). They showed a clear core-periphery structure with multiple peripheral nodes/subgroups. The core transitioned to the level of network administration. As it can be seen in network C, the type of nodes in the network core changed from intra-organisational to inter-organisational collaborations and included nodes from other OnkoAktiv networks (e.g. core of network C: C01, C00, C15, A00, A07, A08, A09, A28, F00, G00, H00, E00, D00, M00, B00). Further, the core-actors in network A were organized as neutral administrative body to administrate the network. However, we must point out that none of the OnkoAktiv networks had fully reached the highest, integrated stage (“new service”) of organizational forms yet.

## Discussion

The network OnkoAktiv aims to integrate exercise services into the German cancer care system. Thus, OnkoAktiv builds regional “sub-networks” that connect health care professionals (HCPs) and actors from different relevant fields with cancer-specific exercise programs to provide a comprehensive exercise provision for cancer patients. To date, there is only little knowledge about the individual OnkoAktiv network composition. Therefore, the objective of this study was to analyze the current network structures of 11 OnkoAktiv networks in regard to their major characteristics and collaborative structures. Further, they have been classified into their stage of organizational forms and implications for further network development in the field of exercise oncology have been made. In the following, we first discuss the structural characteristics the analysis revealed, before describing the organisational forms and practical implications for OnkoAktiv.

### Node and tie attributes: what are the tasks and professions of the network actors?

Our analysis indicated that smaller networks included more similar professions than larger networks. Interestingly, even smaller networks with less than 20 nodes involved at least four and up to nine medical professions into their network. Larger networks spanned their ties into other operational fields and showed a greater network diversity. Economic researchers examined different types of group diversity and their advantages. One type of diversity has been described as “variety” that assumes that members within units differ from one another with regard to categorical attributes such as functional background or expertise. Diversity on categorical attributes is associated with greater creativity, innovation, increased flexibility, better decision-making processes and firm performance [27–29]. Different researchers examined that collaboration in healthcare appears more likely on the horizontal, rather than across units (vertical) [30, 31]. OnkoAktiv aims to encourage HCPs and exercise professionals to collaborate across professional fields to provide the highest value of exercise care for cancer patients.

### Cohesion: number of nodes and ties, average degree

OnkoAktiv networks presented a high range in network size, number of ties and average degree. High average degree has been indicated with higher trust and perceived value in public health collaborations [32]. However, high average degree could also result in redundancies of relations. The creation and sustainability of any relation needs ego´s resources, like time and personnel, which is why some relations might deliver barely any advantages. The development of new network relations is only reasonable if the potential new network actor holds different and not yet existent resources. Further, one scarce resource in OnkoAktiv networks are relations into the financial sector. Interestingly, several community-based exercise program evaluations highlighted that long-term funding and cost coverage have been major problems in exercise program implementation [33–35].

### Centrality: who are actors in the center of the network?

The analysis of degree centrality illustrated that central actors (besides ego) with high degree centrality were highly important and held mostly influential positions like clinical directors or leading oncologists. Those influential actors can inhibit or foster the creation of social capital by linking multiple other important actors from different fields to their network [36]. Moreover, central nodes might be important “change-makers” that control power and the flow of information or resources [37, 38]. Long argues that important actors with high degree centrality can play the role of initiators or a natural governmental body [39]. OnkoAktiv coordinators showed high betweenness centrality and owned high influence on network flows as brokers between different parts of the network. They supported inter-organisational collaborations by linking actors from different social fields [36–38, 40].

### Organisational forms in exercise oncology: implications for the network OnkoAktiv

The studied OnkoAktiv networks could be classified into a continuum of organisational forms that is based on the work by Leutz [25]. OnkoAktiv networks, defined as “linkage”, showed some ties between medical professionals and the OnkoAktiv coordinator who acts as bridging component. In this early stage of organisational forms, network coordinators should start with an analysis of potential professional leaders, their relational structure, the formation of individual benefits and usefulness of network membership for each actor [24, 25]. They should promote and support communication between potential network members [41]. This strategy corresponds to “diffusion of innovations” theory as described by Rogers [42], considering the “knowledge” and “persuasion”-stages of potential members as crucial for project implementation. Accordingly, the decision-making process of participation is an “information-seeking” process in which potential participants increase their persuasion about membership advantages. Networks categorised as “linkage” prosper through individual engagement, perceived personal value, knowledge-exchange and service to service engagement. Based to the ACSM guidelines [6], professional linkage provides the opportunity to refer cancer patients from service (e.g. primary care) to service (exercise programs). In this stage, HCPs should assess cancer- and cancer-therapy-related symptoms (that can be diminished by exercise), current exercise level, interests and possible contraindications to exercise. If they characterise patients in level 1 as „universal” they advise to increase or maintain physical activity levels and refer their cancer patient directly into public CBEP.

Further network development may result in a transition into more integrated, co-ordinated networks. Several recent studies analysed hospital networks in health care as co-ordinated networks [12, 43, 44]. In our study, we classified networks H and J in this stage because horizontal and vertical links have been created between organisational units. Such collaborations enhance productivity, intellectual content and the creation of new forms of resources [32, 45]. Although, collaboration may come with challenges on different levels, like lack of staff, time and structural resources, low motivation, hindered goal consensus or agreement of decision-making [32, 46, 47]. In this stage, OnkoAktiv coordinators need to support a shared vision and clearly define the network mission statement. They coordinate benefits, manage the sequence of services, share clinical information within a planned framework and manage patient transitions between services [25]. Co-ordinated networks can handle patients in level 2 “targeted”. They should get referred by HCPs into cancer-specialized exercise programs or to OnkoAktiv coordinators, who enable further assessments and exercise consultations to clear up exercise safety [6].

The transition into the highest form of integrated networks has been discussed from different perspectives in research [23–25]. It is important to underline that full integration of health services does not have to be the optimal outcome. Based on the work by Lawrence and Lorsch [48], the level of integration should be connected to the degree of specialization of healthcare services that is needed to serve the individual patient. The higher the need for specialization, the greater the demand of integration. What we have seen in OnkoAktiv networks is, that in larger, more integrated networks, the core of the network worked as administrative body, connecting the local network to others across regions. Further, the core connected many oncological specialists within a core-periphery-structure that was able to serve highly vulnerable patients as case management. However, the more integrated OnkoAktiv networks should be also able to serve universal patients and support direct linkage between HCPs and exercise programs to prevent oversupply of services. As seen in Fig. 1, before starting any exercise intervention, cancer patients need to get screened and classified into their level of complexity, individually cleared up to exercise and then referred into the appropriate exercise care program. Table 3 summarizes the operations for OnkoAktiv coordinators in different levels of organisational network forms based on our argumentation. In our framework, a network can transit into a higher level of network forms when all operations of the lower stage have been accomplished.

**Table 3:** Summary of network classification parameters and operations for OnkoAktiv coordinators in different stages of network forms

Operation	Linkage	Co-ordination	Full Integration
Function of coordinators	Initiation of collaboration between stakeholder and professional leader, support of communication and share of knowledge. Definition of network vision and mission statement Clarify and define jointly individual tasks, responsibilities and proposals of each network member	Administration of network members, align network goals to “core business” of network members Support shared vision, keep individual responsibilities in subgroups, create joints for collaboration and a “platform” for communication (e.g. regular meetings)	Provision of all care services as integrated care model. Develop a new administrative system and a shared vision and mission statement. Implement pooled funding options
Key player involvement	Analysis of “core”-actors and important key player inside the organisation: Involvement of clinical managers, heads of exercise or oncology department, leading clinical nurses and oncologists	Analysis of important and influential “peripheral”-actors inside and outside the organisation Inside: e.g. physio- and exercise therapy, urology, haemato-oncology, gastroenterology or breast care centers Outside: Involvement of health and annuity insurance, self-help groups or cancer care society	Fully integrate key player into a “new organisation” with multidisciplinary teams
Funding	Individual/separate	Individual/separate	Individual/separate or pooled
Patients need	Universal	Targeted	Specialist

### Limitations

Our study was limited by the number of OnkoAktiv networks and the openness of OnkoAktiv coordinators to share information about their relations. We had no access to the collaborative stakeholders for interviews to enable a full network analysis, which is why we applied an ego-centric network analysis. However, using centrality parameters in ego-centric networks analyses might be biased by ego’s subjective perspective. As it has been discussed by Borgatti [49] as well as Henderson [50], the ethical base of SNA can be restrictive or denying due to vulnerable work-related information. Further, the subjective estimation of ties between actors could be incomplete and missing data may result from oblivion or response-fatigue. Further, the definition of network boundaries has been challenging because of overlapping fields of activities and dynamic work-relationships. Our results should be interpreted with caution because of a missing proof of correctness of ego´s answers [51]. The practical implications (see Table 3) only represent cross sectional, descriptive data and allow no generalization.

While network governance (or management) was not an explicit aspect of this study, further research should consider network governance modes [52] with regard to effective network management and how this might contribute to a better exercise integration into cancer care.

## Conclusion

This work aimed to better understand the collaborative network structures of OnkoAkiv and to define specific tasks for further network development. We could classify each network into their developmental stage of organizational forms and highlighted specific network characteristics for each OnkoAktiv network. Based on our analysis, we developed operations for network coordinators in different stages of network forms to enhance their local networks.

Collaborative networks enable the integration of pathways of exercise care into oncology that involve stakeholder from different medical and social fields. While there is little research about exercise integration into oncology settings, the network OnkoAktiv provides an example of relevant actors such as oncologists, sport scientists or clinical directors can be connected for a comprehensive exercise provision. However, more longitudinal studies are required to examine network maturity and activities that influence network outcomes such as efficiency.

## Supplementary Information

**Additional file 1:** **Supplement 1.** Analysis of medical professions in number of actors and percentages per network.

## Data Availability

The datasets used and/or analysed during the current study are available from the corresponding author on reasonable request.

## Abbreviations

WHO: World Health Organisation
SNA: Social network analysis
HCP: Health care practitioners
NAO: Network administrative organisation
M: Mean value
SD: Standard deviation
N: Number
Avg: Average
