# Supplementary material for: Exploring the organisational structure of networks for exercise oncology provision: a social network analysis of OnkoAktiv
Source: BMC Health Serv Res. 2023 May 27;23:555. doi: 10.1186/s12913-023-09572-8 (PMC10225106; doi:10.1186/s12913-023-09572-8)
Supplement: Supplementary file 1 — Additional file 1: Supplement 1. Analysis of medical professions in number of actors and percentages per network. [file 12913_2023_9572_MOESM1_ESM.docx]

**Exploring the organisational structure of networks for exercise oncology provision:**
A social network analysis of OnkoAktiv

Authors:

Annelie Voland; Maximilian Köppel; Stefan Peters; Joachim Wiskemann; Hagen Wäsche

Supplement 1: Analysis of medical professions in number of actors and percentages per network

| **Networks** |  | **G** | **I** | **L** | **M** | **D** | **H** | **J** | **E** | **B** | **C** | **A** |
| --- | --- | --- | --- | --- | --- | --- | --- | --- | --- | --- | --- | --- |
| Total # of nodes by network | [n] | 12 | 16 | 17 | 19 | 19 | 23 | 26 | 32 | 33 | 39 | 52 |
| Exercise science/sports medicine | [n] | 5 | 3 | 6 | 5 | 5 | 7 | 2 | 12 | 11 | 18 | 15 |
|  | [%] | 42 | 19 | 35 | 26 | 26 | 30 | 8 | 38 | 33 | 46 | 29 |
| Oncology/Haemato-oncology | [n] | 1 | 3 | 3 | 2 | 3 | 2 | 5 | 1 | 5 | 7 | 7 |
|  | [%] | 8 | 19 | 17 | 11 | 16 | 9 | 19 | 3 | 15 | 18 | 14 |
| Clinical director | [n] | 0 | 1 | 2 | 4 | 0 | 1 | 1 | 0 | 1 | 2 | 4 |
|  | [%] | 0 | 6 | 12 | 21 | 0 | 4 | 4 | 0 | 3 | 5 | 8 |
| Gynecology | [n] | 0 | 0 | 1 | 1 | 1 | 3 | 2 | 1 | 2 | 0 | 1 |
|  | [%] | 0 | 0 | 6 | 5 | 5 | 13 | 8 | 3 | 6 | 0 | 2 |
| Rehabilitation | [n] | 2 | 0 | 0 | 0 | 0 | 0 | 0 | 2 | 0 | 3 | 0 |
|  | [%] | 17 | 0 | 0 | 0 | 0 | 0 | 0 | 6 | 0 | 8 | 0 |
| Physiotherapy/Ergotherapy | [n] | 0 | 0 | 0 | 1 | 2 | 1 | 2 | 0 | 2 | 0 | 0 |
|  | [%] | 0 | 0 | 0 | 5 | 11 | 4 | 8 | 0 | 6 | 0 | 0 |
| Nursing | [n] | 1 | 0 | 0 | 3 | 0 | 0 | 2 | 1 | 0 | 0 | 0 |
|  | [%] | 8 | 0 | 0 | 16 | 0 | 0 | 7 | 3 | 0 | 0 | 0 |
| Surgery | [n] | 1 | 0 | 0 | 0 | 0 | 0 | 1 | 2 | 0 | 0 | 0 |
|  | [%] | 8 | 0 | 0 | 0 | 0 | 0 | 4 | 6 | 0 | 0 | 0 |
| Orthopaedy | [n] | 0 | 1 | 0 | 1 | 1 | 0 | 1 | 1 | 1 | 0 | 0 |
|  | [%] | 0 | 6 | 0 | 5 | 5 | 0 | 4 | 3 | 3 | 0 | 0 |
| Psycho-oncology | [n] | 0 | 0 | 0 | 0 | 1 | 1 | 0 | 0 | 0 | 1 | 2 |
|  | [%] | 0 | 0 | 0 | 0 | 5 | 4 | 0 | 0 | 0 | 3 | 4 |
| Paediatrics | [n] | 0 | 0 | 0 | 0 | 0 | 1 | 0 | 0 | 1 | 0 | 1 |
|  | [%] | 0 | 0 | 0 | 0 | 0 | 4 | 0 | 0 | 3 | 0 | 2 |
| Urology | [n] | 0 | 0 | 0 | 1 | 1 | 0 | 0 | 0 | 0 | 0 | 0 |
|  | [%] | 0 | 0 | 0 | 5 | 5 | 0 | 0 | 0 | 0 | 0 | 0 |
| Clinical nutrition | [n] | 0 | 0 | 0 | 0 | 1 | 0 | 0 | 1 | 0 | 0 | 1 |
|  | [%] | 0 | 0 | 0 | 0 | 5 | 0 | 0 | 3 | 0 | 0 | 2 |
| Ear-Nose-Throat | [n] | 0 | 0 | 0 | 0 | 0 | 0 | 0 | 0 | 1 | 0 | 0 |
|  | [%] | 0 | 0 | 0 | 0 | 0 | 0 | 0 | 0 | 3 | 0 | 0 |
| Cardiology | [n] | 0 | 0 | 0 | 1 | 0 | 0 | 0 | 0 | 0 | 0 | 0 |
|  | [%] | 0 | 0 | 0 | 5 | 0 | 0 | 0 | 0 | 0 | 0 | 0 |
| Social Service | [n] | 0 | 0 | 0 | 0 | 0 | 0 | 0 | 0 | 0 | 0 | 1 |
|  | [%] | 0 | 0 | 0 | 0 | 0 | 0 | 0 | 0 | 0 | 0 | 2 |
| Neurology | [n] | 0 | 1 | 0 | 0 | 0 | 0 | 0 | 1 | 0 | 0 | 0 |
|  | [%] | 0 | 6 | 0 | 0 | 0 | 0 | 0 | 3 | 0 | 0 | 0 |
| Radiology | [n] | 0 | 1 | 0 | 0 | 0 | 0 | 0 | 0 | 0 | 0 | 1 |
|  | [%] | 0 | 6 | 0 | 0 | 0 | 0 | 0 | 0 | 0 | 0 | 2 |
| Internal medicine | [n] | 0 | 1 | 0 | 0 | 0 | 1 | 1 | 0 | 0 | 0 | 0 |
|  | [%] | 0 | 6 | 0 | 0 | 0 | 4 | 4 | 0 | 0 | 0 | 0 |
| **Nodes in medical sector** | **[n]** | **10** | **11** | **12** | **19** | **15** | **17** | **17** | **22** | **24** | **31** | **33** |
|  | **[%]** | **83** | **69** | **71** | **100** | **79** | **74** | **65** | **69** | **73** | **79** | **63** |
| **Different sectors** |  | **5** | **7** | **4** | **9** | **8** | **8** | **9** | **9** | **8** | **5** | **9** |
